# A Study on the Amelioration of Circadian Rhythm Disorders in Fat Mice Using High-Protein Diets

**DOI:** 10.3390/nu15153459

**Published:** 2023-08-04

**Authors:** Guoliang Deng, Zhiqing Jiang, Hui Lu, Naiyan Lu, Rongxiang Zhu, Chengkai Zhu, Peng Zhou, Xue Tang

**Affiliations:** 1 School of Food Science and Technology, Jiangnan University, Wuxi 214122, China; 6210112142@stu.jiangnan.edu.cn (G.D.); 6200113038@stu.jiangnan.edu.cn (Z.J.); 1012190707@stu.jiangnan.edu.cn (H.L.); lunaiyan@jiangnan.edu.cn (N.L.); 6210113263@stu.jiangnan.edu.cn (R.Z.); 6220111270@stu.jiangnan.edu.cn (C.Z.); zhoupeng@jiangnan.edu.cn (P.Z.); 2 National Engineering Research Center for Functional Food, Jiangnan University, Wuxi 214122, China; 3 Collaborative Innovation Center of Food Safety and Quality Control in Jiangsu Province, Jiangnan University, Wuxi 214122, China; 4 State Key Laboratory of Food Science and Technology, Jiangnan University, Wuxi 214122, China

**Keywords:** high-protein diets, lipid metabolism, circadian rhythm, obesity, transsulfuration pathway

## Abstract

This innovative study investigates the effects of high-protein diets (milk protein) on the circadian rhythm of hepatic lipid metabolism. We aimed to understand how high-protein interventions regulate biological clock genes, maintain lipid metabolism balance, and affect the circadian rhythm of antioxidant levels in vivo. We divided 120 SPF-class C57BL/6J mice into the control, high-fat/low-protein (HF-LP), and high-fat/high-protein (HF-HP) groups. Mice were sacrificed during active (2 a.m. and 8 a.m.) and rest periods (2 p.m. and 8 p.m.). In the HF-LP group, hepatic lipid anabolic enzymes were consistently expressed at high levels, while key lipolytic enzymes slowly increased after feeding with no significant diurnal differences. This led to an abnormal elevation in blood lipid levels, a slow increase in and low levels of superoxide dismutase, and a rapid increase in malondialdehyde levels, deviating from the diurnal trend observed in the control group. However, high-protein interventions in the HF-HP group restored lipid synthase activity and the expression of key catabolic enzymes, exhibiting a precise circadian rhythm. It also improved the lipid-metabolism rhythm, which was disrupted by the high-fat diet. Overall, high-protein interventions restored the expression of key enzymes involved in lipid metabolism, improving the lipid-metabolism rhythm, which was disrupted by the high-fat diet.

## 1. Introduction

With the increase in economic and social developments in recent years, obesity caused by high-fat dietary patterns has become a worldwide public health issue [1]. An increase in obesity has also resulted in an increase in the prevalence of diabetes, hypertension, hyperlipidemia, and other diseases [2]. Epidemiological surveys have shown that a long-term excessive high-fat diet intake can disrupt the balance of energy metabolism and cause lipid accumulation, resulting in obesity or becoming overweight [3,4]. In obese people, the incidences of lipid metabolism disorders [5], imbalances of redox homeostasis [6] in the body, and other metabolic syndromes increase. Circadian rhythms refer to the regular and repeated changes in the vital activities of living organisms with the alternation of time and are divided into the core and peripheral biological clocks [7]. For mammals, sleep, diet, exercise, the endocrine system, lipid metabolism, and other vital activities are controlled by biological clocks [8,9,10]. The liver belongs to the peripheral biological clock and is also the main site of lipid metabolism. The relevant regulators and rate-limiting enzymes of hepatic lipid metabolism are regulated by biological rhythms [11]. The biological clock controls the genes for energy utilization [12]. By contrast, a high-fat diet affects the expression of genes of the hepatic peripheral biological clock, leading to an imbalance of the redox state [13,14], and causing rhythmic disorders of lipid metabolism [15,16].

The high-protein diet mouse model is an intervention model based on body weight regulation through macronutrient control, which is defined as a protein supply outweighing 15% of the energy supply ratio [17]. Currently, a long-term high-protein diet is a more effective measure to control obesity and maintain muscle mass than exercise, energy restriction, and ketogenic dietary patterns [18,19,20]. High-protein dietary patterns play an important role in improving lipid metabolism, protein metabolism, and redox status. In particular, a high-protein diet can inhibit lipid synthesis, improve protein synthesis, maintain muscle mass, improve energy expenditure, reduce fat formation and liver fat accumulation, and reduce body weight and body fat percentage [21]. Additionally, it can enhance satiety [22] and reduce energy intake, as well as regulate intestinal flora and the secretion of hormones related to lipid metabolism, such as insulin [23,24], ultimately leading to weight loss. Studies have shown that high-protein dietary interventions downregulate genes related to hepatic de novo lipid synthesis and upregulate genes related to lipid catabolism [25,26], which may improve lipid metabolism disorders caused by high-fat diets. There are few reports on whether high-protein dietary interventions regulate circadian rhythm disorders associated with obesity. Interestingly, studies have shown that dietary metabolites such as amino acids like tryptophan [27] can reset circadian rhythms. This provides theoretical support for the idea that high protein levels may reverse circadian rhythm disorders caused by a high-fat diet. We aimed to find out whether a high-protein dietary intervention regulates biological rhythms in obese mice, and we observed improvements in lipid metabolism and redox status.

## 2. Materials and Methods

### 2.1. Animal Experimental Design and Grouping

One-hundred and twenty male SPF-grade C57BL/6 mice (4 weeks old, initial weight 18.0 ± 0.6 g) were purchased from Shanghai Shrek Laboratory Animal Co., Ltd. (Shanghai, China). All operations and processing of all the animal experiments were carried out in strict compliance with the working rules of the Laboratory Animal Management Committee of Jiangnan University (animal breeding license number: JN.NO20151118-20160511, per the Guidelines for Ethical Review of the National Laboratory Animal Welfare). The animals were raised in a standard barrier environment, with an ambient temperature of 23 ± 2 °C, a humidity of 60 ± 5%, and a light/dark cycle of 12/12 h (8:00–20:00). Throughout the experiment, the animals were allowed free access to food and water.

Milk protein concentrate (MPC) was used as a protein source for the mice. After a two-week accommodation period, the mice were randomly divided into three groups: control (CON; 10% fat, 17% protein), high-fat/low-protein (HF-LP; 44% fat, 17% protein), and high-fat/high-protein (HF-HP; 44% fat, 35% protein). Mice feed formulations are shown in Table 1. Each gradient was then further subdivided into four groups (*n* = 10) according to the time of execution, 8:00, 14:00, 20:00, and 2:00. The body weight and the feed and water intake of the rats were measured once a week.

### 2.2. Tissue Collection of Experimental Animals

After the experiment, the mice were euthanized at four different time points (8:00, 14:00, 20:00, and 2:00 h) after fasting for 12 h. The collected blood was centrifuged in a heparin sodium anticoagulant tube (4 °C, 4000× *g*, 10 min) to obtain the plasma, which was then stored at −80 °C for future use. Liver tissue samples (0.1 g) were fully ground in precooled normal saline to obtain a 10% (*v*/*v*) homogenate, which was centrifuged at −80 °C (4 °C, 3000× *g*, 10 min). To prepare RNA samples, 0.1 g of tissue was placed in 1 mL of precooled TRIzol (Shanghai Biosharp, Shanghai, China).

### 2.3. Plasma and Tissue Biochemical Analysis

According to the instructions of the four blood lipids kit, four blood lipids (triglycerides (TG), total cholesterol (TC), low-density lipoprotein cholesterol (LDL-C), and high-density lipoprotein cholesterol (HDL-C)) were determined, which were purchased from Nanjing Jiancheng Bioengineering Research Institute; additionally, plasma creatinine (CRE) and blood urea nitrogen (BUN) levels were measured. The kit’s instructions utilized the guidelines provided by Nanjing Jiancheng Biotechnology to specify how to determine liver hydrogen sulfide (H2S) levels.

### 2.4. Antioxidant Levels in Plasma and Tissues

The total antioxidant capacity (T-AOC), glutathione peroxidase activity (GSH-PX), glutathione (GSH), oxidized glutathione (GSSG), catalase activity (CAT), superoxide dismutase (SOD), and malondialdehyde (MDA) were measured according to the manufacturer’s instructions (Nanjing Jiancheng Bioengineering Institute, Nanjing, China).

### 2.5. RNA Extraction and Real-Time PCR Analysis

For assessing RNA purity and concentration, we utilized a NanoDrop™ One/OneC Microvolume UV-Vis spectrophotometer (Thermo Fisher Scientific, Waltham, MA, USA), alongside electrophoresis tests to examine RNA quality levels; additionally, we conducted reverse transcription on RNA using an RT-PCR kit (Nanjing Vazyme Biotechnology, Nanjing, China) to generate complementary DNA (cDNA). Then, we combined cDNA with the Q-PCR kit per the manufacturer’s specifications and performed real-time PCR testing (Thunderbird; Fombo Co., Ltd., Osaka, Japan) to amplify the genetic material while detecting fluorescence intensity. An initial cycle of 5 min at 95 °C was carried out, before proceeding onto 45 cycles lasting 20 s each cycle at 95 °C, and 30 s each cycle at 60 °C; the final round involved cycling at 72 °C for one min. To determine gene expression, we leveraged β-actin as a reference material and used the threshold period method. Gene primers were derived using the primer databases NCBI and GenBank, with comprehensive details provided in Table 2. Suzhou GENEWIZ Biotechnology Co., Ltd. (South Plainfield, NJ, USA) assisted with gene primer synthesis.

### 2.6. Statistical Analyses 

Results are expressed as mean ± standard deviation (SD). The results were analyzed using one-way ANOVA followed by Tukey’s multiple-comparisons test, and significance was set at */# *p <* 0.05 and **/## *p <* 0.01. For non-homogeneous variance values, Tamhane’s T2 test was used. Statistical analyses and plotting were performed using GraphPad Prism 8 (GraphPad Software, Inc., San Diego, CA, USA). 

## 3. Results

### 3.1. Effects of a High-Protein Diet on The Growth Performance of Mice

**Figure 1:**
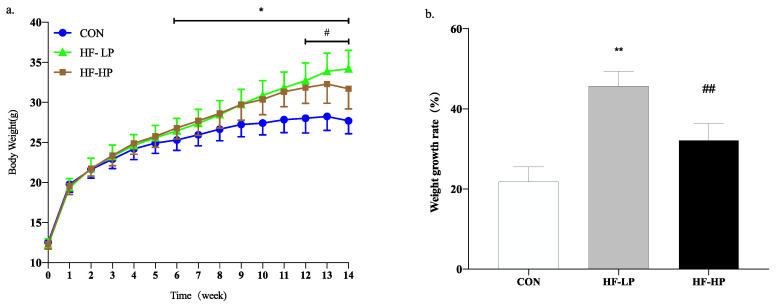
Trends for changes in body weight over 14 weeks in the MPC group (**a**) and weight growth rate (**b**). Values are presented as mean ± SD, *n* = 10 per group. * *p* < 0.05, HF-LP group compared with the CON group; # *p* < 0.05, HF-LP group compared with the HF-HP group, ** *p* < 0.01, HF-LP group compared with the CON group; ## *p* < 0.01, HF-LP group compared with the HF-HP group. MPC, milk protein concentrate; SD, standard deviation; CON, control; HF-LP, high-fat/low-protein; HF-HP, high-fat/high-protein.

To investigate the potential mitigating effects of a high-protein diet on the detrimental effects of a high-fat diet, we first assessed trends in the body weight of mice after they were subjected to different feeding protocols for 14 weeks. At week 6, body weight dramatically increased in the HF-LP group compared with the CON group and decreased significantly at week 12 in the HF-HP group compared with the HF-LP group. Figure 1b shows that the HF-LP group gained 45.73% in body weight, reaching 2.08 times that of the CON group, and that they had a significant reduction in body weight by approximately 32.16% after the high-protein dietary intervention.

It is not difficult to determine whether a high-protein diet can counteract the obesity problems associated with a high-fat diet. We measured typical growth performance indicators in mice to investigate how high protein levels reduce body weight. In Table 3, compared with the HF-LP group, the HF-HP group showed a significant decrease in body fat percentage by approximately 26.23% and a highly significant increase in muscle index by approximately 5.97%; there was no obvious difference in energy intake. Thus, a high-protein diet can reduce body weight by reducing fat content and maintaining muscle mass, indicating that a high-protein diet can alleviate the development of obesity to some extent.

### 3.2. Effects of a High-Protein Diet on Liver and Plasma Markers and Circadian Rhythms

**Figure 2:**
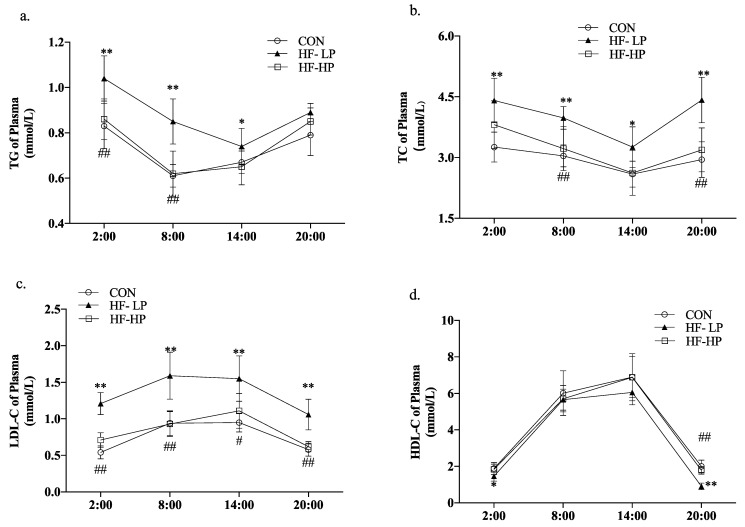
Effects of a high-protein diet on blood lipid levels and circadian rhythm. (**a**) Circadian rhythm variation in plasma TG levels. (**b**) Circadian rhythm variation in plasma TC levels. (**c**) Circadian rhythm variation in plasma LDL-C levels. (**d**) Circadian rhythm variation in plasma HDL-C levels. Data are expressed as the mean ± SD (*n* = 10 rats per group); * *p* < 0.05, HF-LP group compared with the CON group; # *p* < 0.05, HF-LP group compared with the HF-HP group, ** *p* < 0.01, HF-LP group compared with the CON group; ## *p* < 0.01, HF-LP group compared with the HF-HP group. SD, standard deviation; CON, control; HF-LP, high-fat/low-protein; HF-HP, high-fat/high-protein; TC, total cholesterol; LDL-C, low-density lipoprotein cholesterol; HDL-C, high-density lipoprotein cholesterol; TG, triglycerides.

In mice, lipids are usually deposited in the plasma and liver. Studies have shown that circadian rhythms influence lipid levels in mice, and TC, TG, HDL-C, and LDL-C are preliminary indicators of lipid deposition. To investigate the improvement of lipid deposition in mice by restoring circadian rhythms with a high-protein diet, lipid profiles were measured in dark-active mice, and the experimental light time was from 8:00 to 20:00. In this experiment, 20:00 to 2:00 were the active times and 8:00 to 14:00 were the resting times. As shown in Figure 2a,b, in the HF-HP group, the TG and TC levels were significantly lower than those in the HF-LP group, and the circadian rhythm changes were significantly different from those in the CON group. As shown in Figure 2c,d, LDL-C levels in the HF-LP group were apparently higher than those in the CON group. In contrast, HDL-C levels were lower than those in the other two groups; this could be due to dyslipidemia caused by the excessive intake of high-fat chow during the activity period. TG, TC, and LDL-C levels in the HF-HP group returned to levels like those found in the CON group. At 8:00, they were reduced by approximately 37.1%, 22.7%, and 71.0%, respectively, compared with those in the HF-LP group. The circadian rhythm trend of HDL-C levels was similar across all groups, showing no significant differences. This suggests that a high-protein diet has a more significant effect on improving the circadian rhythm variation in LDL-C levels. In conclusion, a high-protein diet can reverse the circadian rhythm disturbances in lipid levels associated with a high-fat diet, leading to a reduction in lipid accumulation in the body.

**Figure 3:**
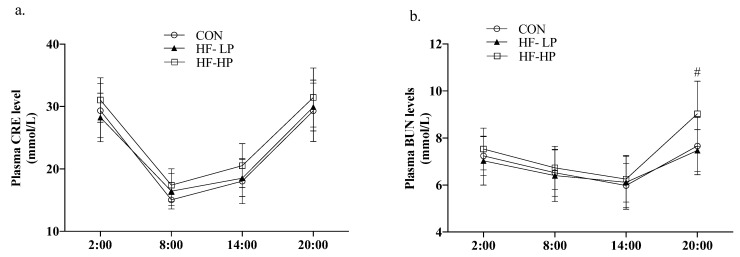
Effect of high-protein diet on circadian rhythm of plasma CRE and BUN level. (**a**) Circadian rhythm variation in plasma CRE level, (**b**) Circadian rhythm variation in plasma BUN level; Data are expressed as the mean ± SD (*n* = 10 rats per group); # *p* < 0.05, HF-LP group compared with the HF-HP group.

Clinical evidence suggests that excessively high protein diets can damage glomerular function. We assessed renal function in the mice by measuring CRE and BUN levels in the Figure 3. Although BUN levels were clearly elevated in the HF-HP group compared with the CON group at 20:00 (*p* < 0.05), there was no evident difference in CRE at any time, most likely due to the low water intake and high amino acid metabolism in mice resting during the day. This indicated that the 35% high-protein diet had essentially no effect on renal function.

### 3.3. Effects of a High-Protein Diet on the Circadian Rhythm of Liver Lipid Metabolism Genes

**Figure 4:**
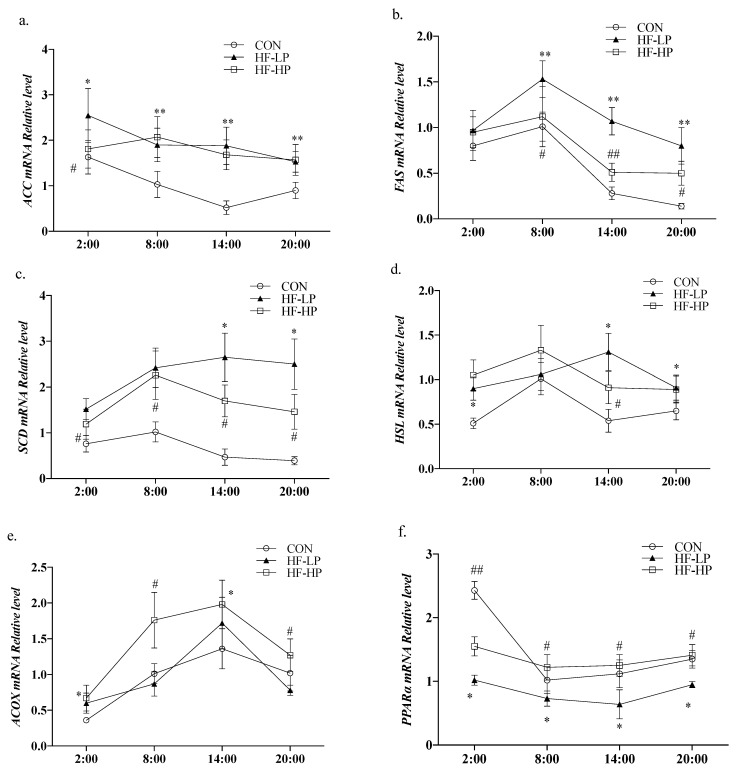
Effects of a high-protein diet on the circadian rhythm changes in liver lipid metabolism genes. (**a**) Circadian rhythm variation in hepatic *ACC* relative levels. (**b**) Circadian rhythm variation in hepatic *FAS* relative levels. (**c**) Circadian rhythm variation in hepatic *SREBP-1C* relative levels. (**d**) Circadian rhythm variation in hepatic *HSL* relative levels. (**e**) Circadian rhythm variation in hepatic *ACOX* relative levels. (**f**) Circadian rhythm variation in hepatic *PPARα* relative levels. Data are expressed as the mean ± SD (*n* = 10 rats per group); * *p* < 0.05, HF-LP group compared with the CON group; # *p* < 0.05, HF-LP group compared with the HF-HP group, ** *p* < 0.01, HF-LP group compared with the CON group; ## *p* < 0.01, HF-LP group compared with the HF-HP group. SD, standard deviation; CON, control; HF-LP, high-fat/low-protein; HF-HP, high-fat/high-protein; *ACC*, acetyl coenzyme A carboxylase α; *FAS*, fatty acid synthase; *SREBP-1C*, sterol regulatory element binding protein 1C; *ACOX1*, acyl coenzyme A oxidase 1; *HSL*, hormone-sensitive lipase; *PPARα*, peroxisome proliferator-activated receptor α.

In Figure 4a,b, acetyl coenzyme A carboxylase α (*ACC)* and fatty acid synthase (*FAS*) expression levels in the CON group were elevated during the active phase and decreased during the resting phase, showing significant diurnal differences. The HF-LP diet significantly increased the expression of *ACC*, *FAS*, and sterol regulatory element binding protein 1C (*SREBP-1C*). As shown in Figure 4d–f, hormone-sensitive lipase (*HSL*) and acyl coenzyme A oxidase (*ACOX*) expression in the HF-LP group increased slowly, indicating that high-fat feeding was unable to metabolize the remaining fat in a timely manner; in contrast, *ACOX* and peroxisome proliferator-activated receptor α (*PPAR-α*) expression was significantly elevated after HF-HP diet intervention (*p* < 0.01), in line with the trend observed in the CON group.

### 3.4. Effects of a High-Protein Diet on the Circadian Rhythm of Redox Status in Mice

**Figure 5:**
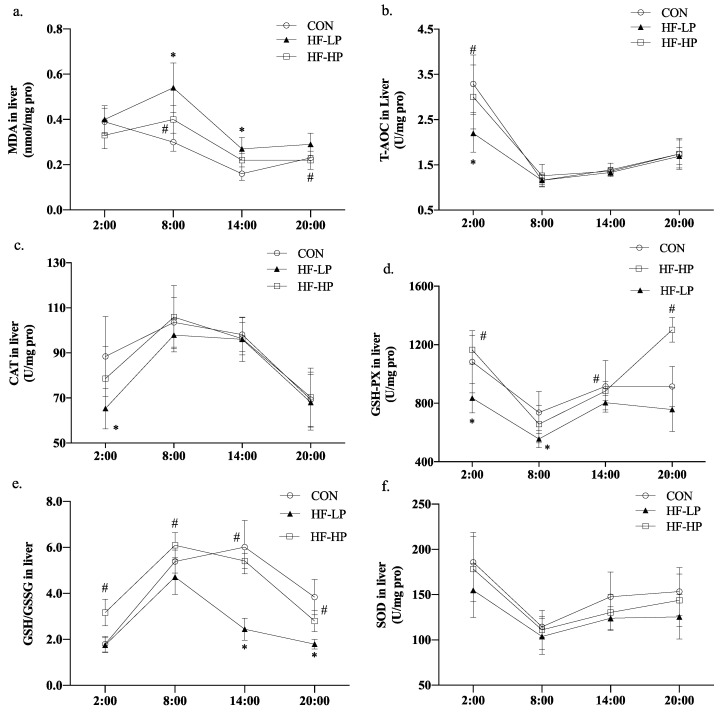
Effects of a high-protein diet on the circadian rhythms of liver redox status. (**a**) Circadian rhythm variation in hepatic MDA levels. (**b**) Circadian rhythm variation in hepatic T-AOC levels. (**c**) Circadian rhythm variation in hepatic CAT levels. (**d**) Circadian rhythm variation in hepatic GSH-PX levels. (**e**) Circadian rhythm variation in hepatic GSH/GSSG levels. (**f**) Circadian rhythm variation in hepatic SOD levels. Data are expressed as the mean ± SD (*n* = 10 rats per group); * *p* < 0.05, HF-LP group compared with the CON group; # *p* < 0.05, HF-LP group compared with the HF-HP group. SD, standard deviation; CON, control; HF-LP, high-fat/low-protein; HF-HP, high-fat/high-protein; H2S, liver hydrogen sulfide; MDA, malondialdehyde; T-AOC, total antioxidant capacity; CAT, catalase activity; GSH-PX, glutathione peroxidase activity; GSH, glutathione; GSSG, oxidized glutathione; SOD, superoxide dismutase.

We investigated whether weight loss due to a high-protein diet affected the redox status of the body. Figure 5a shows that MDA levels in the HF-LP group increased from 2:00 to 8:00, indicating a disrupted circadian rhythm. A high-protein intervention reduced MDA levels. As shown in Figure 5b–d, T-AOC, CAT, and GSH-PX declined in the HF-LP group compared with the CON group but increased in the HF-HP group, and the GSH-PX levels in the HP group returned to the same level as those in the CON group. In Figure 5e, the GSH/GSSG levels in the HF-HP group were evidently higher than those in the HF-LP group at each time point; Figure 5f shows that the SOD levels in the HF group had no significant change than those in the CON group. A high-fat diet disrupted the circadian rhythm of the redox state, and the antioxidant levels were lower than those in the CON group at all time points. The high-protein intervention restored them to near-normal or even better levels than those in the CON group.

### 3.5. Effects of a High-Protein Diet on the Circadian Rhythms of Hepatic H2S Levels and Transsulfuration Pathway-Related Gene Expressions

**Figure 6:**
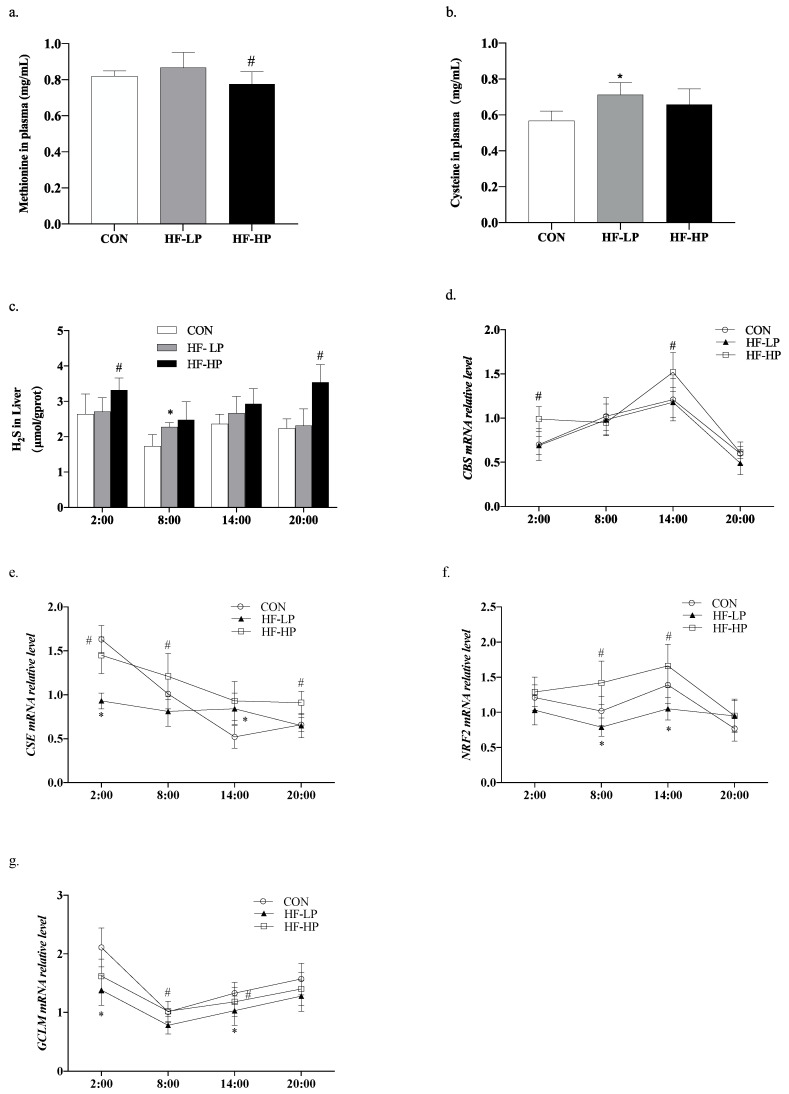
Effects of a high-protein diet on the circadian rhythms of hepatic H2S levels and the transsulfuration pathway-related genes. (**a**) Cysteine levels in plasma. (**b**) Methionine levels in plasma. (**c**) Circadian rhythm variation in hepatic H2S levels. (**d**) Circadian rhythm variation in hepatic *CBS* relative levels. (**e**) Circadian rhythm variation in hepatic *CSE* relative levels. (**f**) Circadian rhythm variation in hepatic *NRF2* relative levels. (**g**) Circadian rhythm variation in hepatic *GCL* relative levels. Data are expressed as the mean ± SD (*n* = 10 rats per group); * *p* < 0.05, HF-LP group compared with the CON group; # *p* < 0.05, HF-LP group compared with the HF-HP group. SD, standard deviation; CON, control; HF-LP, high-fat/low-protein; HF-HP, high-fat/high-protein; *CBS*, cystathionine-beta-synthase; *CSE*, cystathionine-γ-lyase; *NRF2*, nuclear factor erythroid 2-related factor 2; *GCL*M, recombinant glutamate-cysteine ligase modifier subunit.

Methionine can be converted to cysteine under the action of cystathionine-γ-lyase (*CSE*), which is known as the transsulfuration pathway. H2S can regulate the redox state balance [28], and protein-rich methionine generates H2S under the actions of cystathionine-beta-synthase (*CBS*) and *CSE*. To investigate the regulation of the redox state, we checked how H2S influences oxidative stress. As shown in Figure 6a,b, the HF-HP group, which received a diet with 35% protein, produced higher levels of cysteine than the other three groups, indicating rich production of H2S. As shown in Figure 6c, the HF-LP group had increased H2S levels compared with the CON group. This boost could be attributed to compensation brought on by heightened energy metabolism; its modality was converse when weighed against the results seen for mice in the CON group, while the high-protein intervention increased H2S levels by approximately 52.5% compared with that in the HF-LP group at 20:00. As shown in Figure 6d,e, the relative expression levels of liver *CBS* and *CSE* in the HF-HP group were upregulated during the active period and downregulated during the resting period, consistent with the trend in H2S changes. As shown in Figure 6f,g, the relative expression levels of liver nuclear factor erythroid 2-related factor 2 (*NRF2*) increased slowly.

In contrast, the relative expression levels of the recombinant glutamate-cysteine ligase modifier subunit (*GCLM*) were not significantly upregulated during the active phase in the high-fat group. Meanwhile, the relative expression levels of *NRF2* and *GCLM* were downregulated in the HF-LP group compared with those in the CON group, and the relative expression levels of *NRF2* and *GCLM* were upregulated in the HF-HP group compared with those in the HF-LP group; this trend was in line with that of the CON group. This indicated that active *CBS* and *CSE* in mice produce more H2S to regulate *NRF2* and *GCLM* expression, which produces more GSH and alleviates oxidative stress.

### 3.6. Effects of a High-Protein Diet on the Circadian Rhythm Changes in Liver CLOCK Genes and Lipid Metabolism Genes

**Figure 7:**
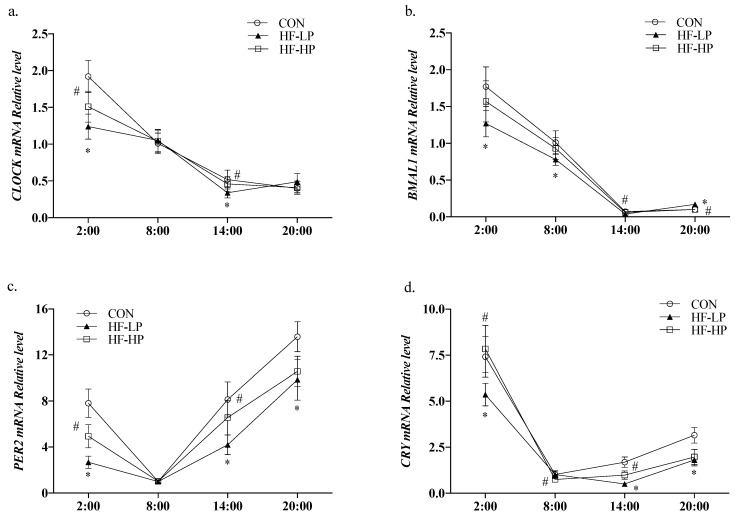
Effects of a high-protein diet on the circadian rhythm changes of liver CLOCK genes. (**a**) Circadian rhythm variation in hepatic *CLOCK* relative levels. (**b**) Circadian rhythm variation in hepatic *BMAL1* relative levels. (**c**) Circadian rhythm variation in hepatic *PER2* relative levels. (**d**) Circadian rhythm variation in hepatic *CRY* relative levels. Data are expressed as the mean ± SD (*n* = 10 rats per group); * *p* < 0.05, HF-LP group compared with the CON group; # *p* < 0.05, HF-LP group compared with the HF-HP group. SD, standard deviation; CON, control; HF-LP, high-fat/low-protein; HF-HP, high-fat/high-protein; *CLOCK*, circadian locomotor output cycle kaput; *BMAL1*, brain and muscle Arnt-like protein 1; *PER2*, period 2; *CRY*, cryptochrome.

Circadian locomotor output cycle kaput *(CLOCK)* and brain and muscle Arnt-like protein 1 (*BMAL1*) are essential components of the core feedback loop that regulates the levels of lipid metabolism genes in the liver. To determine whether a high-protein diet causes changes in the CLOCK genes that modify lipid metabolism, we detected their relative expression levels. As shown in Figure 7a,b, the relative expression levels of *CLOCK* and *BMAL1* in the CON group were high during the active phase and low during the resting phase. The diurnal differences in *CLOCK* and *BMAL1* expression levels were reduced in the HF-LP group, whereas the high-protein intervention brought *CLOCK* and *BMAL1* expression and circadian rhythm higher than in the CON group. As shown in Figure 7c,d, high-fat feeding significantly reduced *PER2* (period2) and *CRY* (cryptochrome) expression levels (*p <* 0.01), and the differences in the changes at each time point were reduced; in contrast, the high-protein intervention resulted in *PER2* and *CRY* expression levels that were closer to those in the CON group. This indicates that a high-protein intervention alleviates liver CLOCK gene expression disruption caused by a high-fat diet or obesity.

## 4. Discussion

High-protein diets (milk protein) are popular for weight loss, reducing body fat percentage, and decreasing visceral fat accumulation [22,29,30]. The regulation of energy metabolism relies on circadian rhythms, mainly controlled by peripheral biological clocks like the liver. These clocks can regulate blood lipid levels, antioxidant capacity, and the expression levels of genes related to lipid metabolism [31,32]. In this study, the expression levels of *ACC*, *FAS*, and *SREBP-1C* (a protein involved in lipid metabolism) remained high during both active and rest periods, while the rapid increase in *HSL* expression after feeding was significantly reduced. This disturbance in circadian rhythms led to the accumulation of fat that could not be effectively broken down, resulting in abnormal blood lipid levels; in contrast, when rats were subjected to a high-protein diet intervention, blood lipid levels decreased significantly. This intervention also upregulated the expression of genes involved in lipid synthesis and downregulated the expression of genes involved in lipid catabolism; consequently, the circadian difference returned to approximately normal levels, indicating that the high-protein intervention effectively regulated metabolic processes and reversed the circadian rhythm disturbance caused by the high-fat diet.

A high-protein diet and substituting methionine with cysteine were shown to regulate antioxidation and produce H_2_S. As a result, the methionine levels in the HF-LP group decreased. The body’s antioxidant capacity is closely linked to ROS levels, [33,34] and H2S can eliminate ROS [35] by reacting directly with H_2_O_2_ or promoting the production of endogenous antioxidants. In this study, we observed that the release of the gaseous regulatory molecule H2S from the livers of mice in the CON group increased during the active phase and decreased during the resting phase. Towards the end of the active phase, this decrease occurred more rapidly. This pattern may be associated with the transportation of H2S to other sites to regulate the balance of redox status in vivo [28], which aligns with the observed trend in antioxidant capacities, such as T-AOC and CAT. In contrast, the HF-LP group exhibited a decrease in liver H2S levels during the active phase and an increase during the resting phase. These changes remained relatively stable across the different time points.

However, T-AOC, CAT, SOD, and GSH-PX showed a slow increase after ingestion, possibly indicating a compensatory upregulation of redox homeostasis to maintain a high-energy metabolic state. The liver consistently exhibited higher H2S levels following the high-protein intervention, corresponding to increased antioxidant indices such as T-AOC, CAT, GSH-PX, GSH/GSSG, and SOD. Additionally, there was a reduction in MDA production in vivo, and the observed change in trends between time points was similar to that in the CON group. These findings suggest that the high-protein diet intervention significantly elevated H2S levels and increased T-AOC, CAT, SOD, GSH-PX, and GSH/GSSG in mice. This indicates that a high-protein intervention may alleviate oxidative stress caused by a high-fat diet by maintaining rhythmic homeostasis of H2S levels.

H2S regulates the *CLOCK–BMAL1* heterodimer to ensure stable expression of *NRF2-GCL*, improving antioxidative ability and maintaining lipid metabolism balance. The liver’s biological clock can control the expression or activity of metabolic enzymes, signaling molecules, and transporter proteins, such as leptin, insulin, and corticosterone, which all exhibit circadian oscillations [36,37]. *CLOCK*, *BMAL1*, and their repressors *CRY* and *PER2* form a core self-feedback regulatory loop [38,39]. The two most important negative feedback loops are as follows:

1. *CLOCK* and *BMAL1*, which bind to form a *CLOCK–BMAL1* heterodimer and regulate peroxisome proliferator-activated receptor α (*PPARα*); this complex trans-activates *PPARα*, which controls the expression of the lipolytic target genes *HSL* and *ACOX* [40,41] that are involved in regulating lipid metabolism homeostasis. *PPARα* positively regulates its transcription by binding to the *PPARα* response element on the *BMAL1* gene promoter [42]; in contrast, *PER2* and *CRY* proteins can interact directly with *CLOCK* and *BMAL1* to inhibit *CLOCK–BMAL1* heterodimer activity and suppress *PER2* and *CRY* transcription, thus completing the self-negative feedback regulatory loop [43,44];
2. The BMAL1 promoter is associated with retinoic acid-related orphan receptor α (*RORα*) and the orphan nuclear hormone receptor α (reverse viral erythroblastosis oncogene product (*REV-ERBα*). These bind to the ROR response element, activating and blocking *BMAL1* transcription. The nuclear receptor *REV-ERBα* inhibits the expression of insulin-induced gene 2 (*INSIG2*), which deregulates the shearing and activation of the lipid metabolism transcription factor SREBP-1C to maintain lipid droplet morphology [45,46]; additionally, *SREBP-1C* promotes the expression of *ACC* and *FAS* [47], key enzymes for the de novo synthesis of fatty acids. Furthermore, the activation of *PPARα* by fatty acids contributes to its involvement in the circadian rhythm of fatty acid oxidative metabolism.

In this study, we found that the relative expression levels of *CLOCK*, *BMAL1*, *CRY,* and *PER* in the high-fat group showed reduced variation at each time point compared with those in the CON group; however, the high-protein intervention reversed this trend and brought the expression levels closer to those in the CON group. This suggests that the feedback regulatory loop involving *CLOCK* and *BMAL1* in high-fat diet mice is less sensitive to circadian changes, resulting in the delayed expression of biological clock genes. High-protein dietary interventions can effectively enhance the transsulfuration pathway to promote H2S production, significantly enhance the rhythmic expression of the clock genes *CLOCK* and *BMAL1*, and alleviate the high-fat diet-induced blunting of clock gene expression; however, the effect on *CLOCK–BMAL1* expression to regulate lipid metabolism genes needs to be further explored. Although this experiment demonstrated the ameliorative effect of cow’s milk protein at the 35% level on circadian rhythm disorders of lipid metabolism in obese mice, it was not explored that other sources of proteins at the same level had similar or better effects, which requires further investigation.

## 5. Conclusions

A high-fat diet results in rhythmic changes in blood lipids, in vivo redox status, liver lipid metabolism, and biological clock genes. Conversely, a high-protein (milk protein) intervention could reverse this disorder, reduce lipid accumulation, and improve lipid metabolism disorders. This study provides a novel research concept by employing a high-protein diet to regulate the hepatic biological clock’s gene expression and homeostasis. By promoting increased H2S production in the transsulfuration pathway, this approach aims to enhance lipid synthesis and catabolic genes.

## Figures and Tables

**Table 1:** Nutrient composition and energy composition of diets.

Composition (g/kg)	CON Group	HF-LP Group	HF-HP Group
Milk protein concentrate	165.0	206.0	412.0
Sucrose	91.1	91.1	91.1
Corn starch	466.1	235.4	29.4
Maltodextrin	125.0	125.0	125.0
Soya bean oil	20.0	20.0	20.0
Lard	20.0	210.0	210.0
Mineral mixture	50.0	50.0	50.0
Vitamin mixture	10.0	10.0	10.0
Cellulose	40.0	40.0	40.0
Sodium carboxymethyl cellulose	10.0	10.0	10.0
Choline bitartrate	2.5	2.5	2.5
Total	1000	1000	1000
Protein/%	17	17	35
Fat/%	10	44	44
Carbohydrate/%	73	39	21

**Table 2:** Real-time quantitative polymerase-chain-reaction primers.

Gene	Upstream Primer (5′−3′)	Downstream Primer (3′−5′)
*β-actin*	GTGACGTTGACATCCGTAAAGA	GCCGGACTCATCGTACTCC
*ACC*	GGCAGCAGTTACACCACATAC	TCATTACCTCAATCTCAGCATAGC
*FAS*	ATGCTGTGGATCTGGGCTGTC	CAGTTTCACGAACCCGCCTC
*HSL*	AGACCACATCGCCCACA	CCTTTATTGTCAGCTTCTTCAAGG
*ACOX*	TCGAAGCCAGCGTTACGAG	ATCTCCGTCTGGGCGTAGG
*SCD*	TCATCCCATCGCCTGCTCTACCC	TGGTGTAGGCGAGTGGCGGAA
*PPAR* *α*	ATGGAGACCTTGTGTATGG	ATCTGGATGGTTGCTCTG
*CBS*	CCAGGCACCTGTGGTCAAC	GGTCTCGTGATTGGATCTGCT
*CSE*	TTCCTGCCTAGTTTCCAGCAT	GGAAGTCCTGCTTAAATGTGGTG
*NRF2*	CTGAACTCCTGGACGGGACTA	CGGTGGGTCTCCGTAAATGG
*GCLM*	AGGAGCTTCGGGACTGTATCC	GGAAACTCCCTGACTAAATCGG
*CLOCK*	AGCACACACACTTCCTCTCTGACAT	ATCAAGGGACTGAACACTCAAGACC
*BMAL1*	AGTCAGATTGAAAAGAGGCGTCG	AGAAATGTTGGCTTGTAGTTTGCTT
*PER2*	TTCTCTGCTGTTCTTGTATCCTTTT	GCTTTCTGCTGGGAGCTAATG
*CRY*	CACTGGTTCCGAAAGGGACTC	CTGAAGCAAAAATCGCCACCT

**Table 3:** Effects of a high-protein diet on body mass, energy intake, liver index, obesity index, and muscle index in mice.

	CON	HF-LP	HF-HP
Final body weight/g	27.70 ± 1.60	34.20 ± 2.30 **	31.70 ± 2.50 ##
Energy intake/(kJ/d)	45.62 ± 1.28	54.18 ± 3.82 **	57.75 ± 3.78
Liver ratio/%	3.42 ± 0.16	3.58 ± 0.23	3.46 ± 0.29
Fat ratio/%	2.70 ± 0.46	4.86 ± 0.98 **	3.85 ± 0.72 ##
Muscle index/%	6.45 ± 0.45	5.98 ± 0.75 **	6.36 ± 0.50 ##

Note: HF-LP compared with the CON group; **, the difference is highly significant *(p* < 0.01). HF-HP compared with the HF-LP group; ##, the difference is highly significant (*p* < 0.01). CON, control; HF-LP, high-fat/low-protein; HF-HP, high-fat/high-protein.

## Data Availability

All the data are available from the first author under reasonable request.
